# Has Authorship in the Decolonizing Global Health Movement Been Colonized?

**DOI:** 10.5334/aogh.4146

**Published:** 2023-06-20

**Authors:** Chris A. Rees, Gouri Rajesh, Hussein K. Manji, Catherine Shari, Rodrick Kisenge, Elizabeth M. Keating, Ikechukwu U. Ogbuanu, Kitiezo Aggrey Igunza, Richard Omore, Karim P. Manji

**Affiliations:** 1 Division of Pediatric Emergency Medicine, Emory University School of Medicine, Atlanta, Georgia, United States of America; 2 Department of Emergency Medicine, Children’s Healthcare of Atlanta, Atlanta, Georgia, United States of America; 3 University of Georgia, Athens, Georgia, United States of America; 4 Department of Emergency Medicine, Muhimbili University of Health and Allied Sciences, Dar es Salaam, Tanzania; 5 Accident and Emergency Department, The Aga Khan Health Services, Tanzania; 6 Emergency Medicine Department, Muhimbili National Hospital-Mloganzila, Dar es Salaam, United Republic of Tanzania; 7 Department of Paediatrics and Child Health, Muhimbili University of Health and Allied Sciences, Dar es Salaam, United Republic of Tanzania; 8 Division of Pediatric Emergency Medicine, University of Utah, Salt Lake City, Utah, United States of America; 9 Crown Agents, Freetown, Sierra Leone; 10 Kenya Medical Research Institute, Center for Global Health Research, (KEMRI-CGHR), Kisumu, Kenya

**Keywords:** decolonization, authorship, global health partnerships

## Abstract

**Background:**

Decolonization in global health is a recent movement aimed at relinquishing remnants of supremacist mindsets, inequitable structures, and power differentials in global health.

**Objective:**

To determine the author demographics of publications on decolonizing global health and global health partnerships between low- and middle-income countries (LMICs) and high-income countries (HICs).

**Methods:**

We conducted a cross-sectional analysis of publications related to decolonizing global health and global health partnerships from the inception of the selected journal databases (i.e., Medline, CAB Global Health, EMBASE, CINAHL, and Web of Science) to November 14, 2022. Author country affiliations were assigned as listed in each publication. Author gender was assigned using author first name and the software genderize.io. Descriptive statistics were used for author country income bracket, gender, and distribution.

**Findings:**

Among 197 publications on decolonizing global health and global health partnerships, there were 691 total authors (median 2 authors per publication, interquartile range 1, 4). Publications with author bylines comprised exclusively of authors affiliated with HICs were most common (70.0%, n = 138) followed by those with authors affiliated both with HICs and LMICs (22.3%, n = 44). Only 7.6% (n = 15) of publications had author bylines comprised exclusively of authors affiliated with LMICs. Over half (54.0%, n = 373) of the included authors had names that were female and female authors affiliated with HICs most commonly occupied first author positions (51.8%, n = 102).

**Conclusions:**

Authors in publications on decolonizing global health and global health partnerships have largely been comprised of individuals affiliated with HICs. There was a marked paucity of publications with authors affiliated with LMICs, whose voices provide context and crucial insight into the needs of the decolonizing global health movement.

## Introduction

Decolonization in global health is a recent movement aimed at relinquishing remnants of supremacist mindsets, inequitable structures, and power differentials in global health practices [1,2]. Colonialist patterns originated from Euro-Western systems and have had far-reaching negative effects in low- and middle-income countries (LMICs), including the stigmatization and discrimination of marginalized and minoritized populations [3]. Recently, there have been numerous efforts to decolonize global health, which include educational efforts, the formation of student interest groups, new curricula in university courses, and calls for leadership shifts, including more equitable representation of LMIC-affiliated individuals on journal editorial boards [3,4,5,6]. However, these efforts have primarily been driven by academic institutions in high-income countries (HICs) [7].

Both preceding and accompanying the recent push to decolonize global health has been widespread recognition among global health practitioners of the need to create equitable partnerships between individuals in LMICs and HICs [8,9]. Equitable global health partnerships may share training, research, and capacity building goals to improve the health of individuals everywhere [10]. Prior publications have proposed guiding principles for equitable global health partnerships aimed at reducing historically inequitable practices in global health collaborations [11,12,13]. The extent to which the proposed development of equitable global health partnerships has been written about by individuals affiliated with LMICs or HICs is unclear.

In original research, multiple studies suggest there is underrepresentation of authors affiliated with LMICs where the published data were collected in a variety of disciplines and geographic locations [14,15,16,17]. Indeed, as much as 15% of publications that have reported work conducted in an LMIC do not have a single author affiliated with the LMIC where the study was conducted [17,18]. However, an assessment of authorship representation in the literature that describes decolonizing global health is lacking. The opinions and lived experiences of individuals in LMICs provide important insight in this long-overdue movement to promote equitable practices in global health.

Here, we aimed to describe the demographics of authors in publications on decolonizing global health and in publications on global health partnerships between LMICs and HICs. We hypothesized that these publications would exhibit underrepresentation of authors affiliated with LMICs and that when LMIC-affiliated authors were included, they would infrequently occupy the most prominent authorship positions (i.e., first and last authors).

## Methods

### Study Design

We conducted a cross-sectional analysis of publications related to decolonizing global health and global health partnerships from the inception of the selected journal databases to the search date of November 14, 2022. This study was exempted from review by the institutional review board at Emory University School of Medicine because no patient data were involved.

### Data Source

In collaboration with a university librarian with expertise in database queries, we iteratively developed a search query that included words including, but not limited to, “decolonize,” “decolonize,” “decolonization,” “decolonization,” “neocolonial,” “neocolonialist,” “partnerships,” “global health,” and “international health” (Appendix). In order to minimize selection bias, we searched several databases with global reach including Medline, CAB Global Health, EMBASE, CINAHL, and Web of Science.

### Inclusion and Exclusion Criteria

Publications, including editorials, viewpoints, review articles, original research articles, or program descriptions related to decolonizing global health or global health partnerships between LMICs and HICs were included. We classified a publication as focused on decolonizing global health if it 1) explicitly discussed this term or 2) described efforts to undo colonial roots in global health. Publications were classified as describing global health partnerships if they 1) explicitly discussed this term, 2) described relationships between health professionals in LMICs and HICs, or 3) described perceived best practices to global health partnerships. Publications were included if they were in the selected databases from the inception of the database to November 14, 2022. No exclusion was made based on the language in which articles were published because we were able to extract all necessary data regardless of the language of the publication. Publications were excluded if they were not related to decolonizing global health or global health partnerships between LMICs and HICs, if they were book chapters, if they were abstracts only and not full publications, or if we could not access the full text.

### Outcomes

Our primary outcome was the proportion of authors of publications on decolonizing global health or global health partnerships that were affiliated with LMICs or HICs. We acknowledge that institutional affiliation may not capture the complexity of an author’s identity, perspective gained from lived experiences, or relationships with LMICs or HICs. However, as these attributes are easily obtainable, we believe they remain useful to assess distribution of voices for the present study.

Secondary outcomes included the authorship order of authors affiliated with LMICs and HICs, the proportion of authors whose names were male or female, the author position of male and female authors, and the proportion of authors who were affiliated with different regions (e.g., Sub-Saharan Africa, East Asia and Pacific, etc.) and country income brackets (e.g., low-income, lower-middle income, etc.) as defined by the World Bank in 2022 [19].

### Data Extraction

We used the software Covidence, which allowed two reviewers (CAR and GR) to independently screen the title and abstract of each publication identified through our query to determine if the publication related to decolonizing global health or global health partnerships. Two investigators (CAR and GR) then independently reviewed the full text of all publications that were deemed relevant through our screening process to remove any publications that did not meet our inclusion criteria. All disagreements regarding the inclusion based on the title, abstract, or full text were discussed with a third investigator who served as an arbiter until consensus was achieved among the investigators.

We developed an electronic data capture form using REDCap to extract all variables of interest from the included publications [20]. Because the included publications appeared in a variety of disciplines (i.e., global health, surgery, pediatrics, etc.) we extracted each journal’s Scimago Journal Rank and their quartile according to Scimago. Scimago is a normalized journal metric designed to allow for comparisons of journal impact across disciplines [21].

### Variables

Two reviewers (CAR and GR) independently extracted the following variables from each included publication: citation, journal name, date of publication (month and year), author first name, author position (e.g., first, second, third, etc.), country affiliation(s) of each author, publication type (e.g., original research, editorial, review, etc.), study country for original research publications, presence of acknowledgements to individuals affiliated with LMICs, and funding source(s). Similar to prior studies [22,23,24,25], we used the software genderize.io to assign probable genders to authors based on their first name [26].

### Statistical Analysis

We used descriptive statistics to estimate the prevalence of authors affiliated with LMICs and HICs, the prevalence of first and last authors affiliated with LMICs and HICs, the proportion of authors whose names were male, female, or indeterminate and their order, and the proportion and distribution of authors affiliated with different countries according to World Bank income brackets and World Bank geographic regions. We plotted the number of publications related to decolonizing global health and partnerships each year. We also explored differences in authorship by income bracket, geographic region, gender, and normalized Scimago journal rank scores of journals that published articles on decolonizing global health and global health partnerships using the Chi-square test. All statistical analyses were conducted using the statistical software package R, version 4.1.2 (R Foundation for Statistical Computing, Vienna, Austria) and figures were created using Microsoft Excel.

## Results

**Figure 1:**
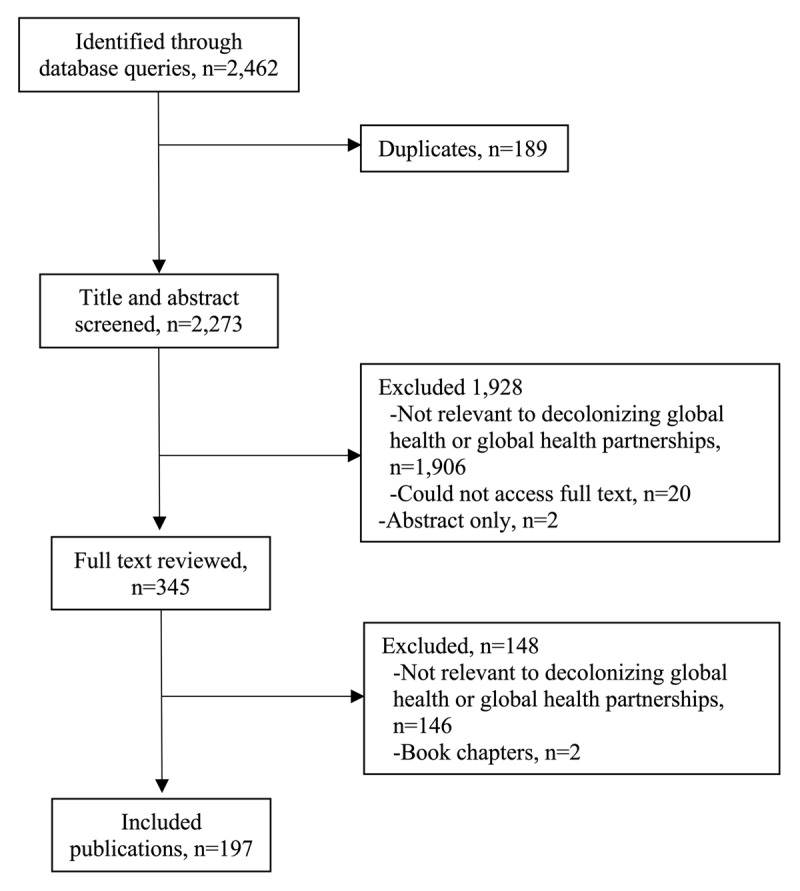
PRISMA Diagram of Included Articles on Decolonizing Global Health and Partnerships Between Low- and Middle-Income Countries and High-Income Countries.

**Figure 2:**
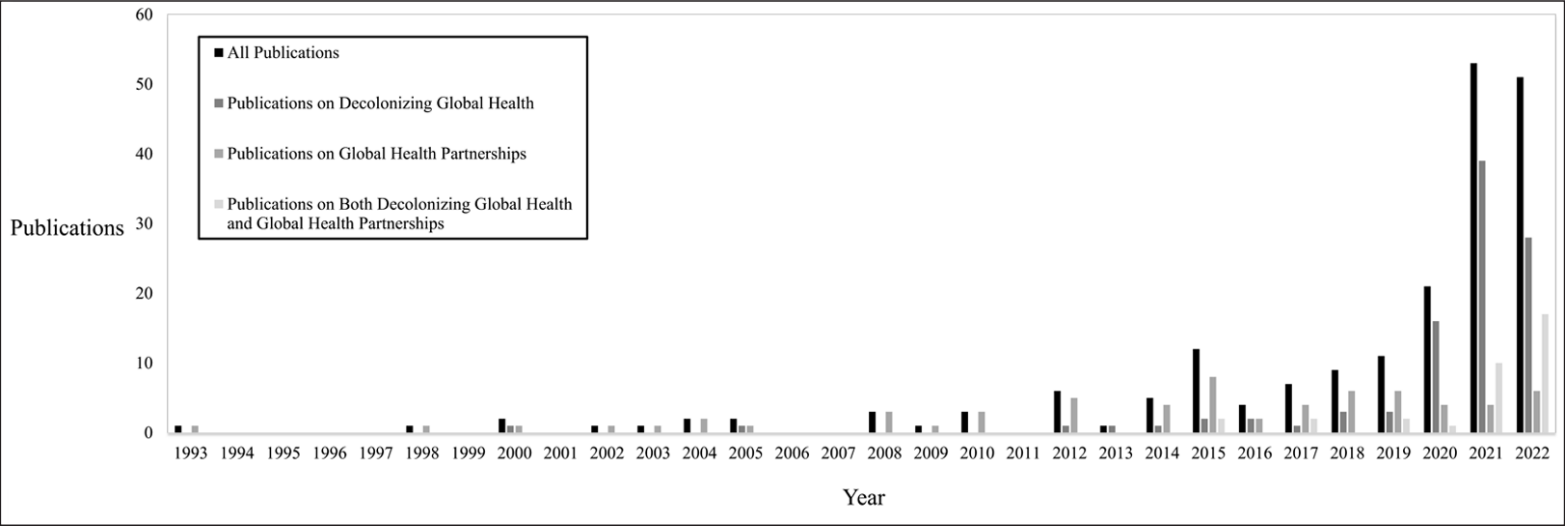
Frequency of Publications on Decolonizing Global Health and Global Health Partnerships by Year of Publication.

There were 2,462 publications identified through our database query and 197 publications met inclusion criteria (Figure 1). The majority (72.0%, n = 138) of the included publications were published in journals ranked in the top quartile according to Scimago Journal Ranking (median 1.003, interquartile range 0.67, 2.263) and most were viewpoint, commentaries, or editorials (89.8%, n = 177) (Table 1). Only 10.2% (n = 20) of the included publications reported original research. Most did not report funding sources (74.6%, n = 147). There was a substantial increase in the number of publications related to global health beginning in the year 2020 (Figure 2). There was a total of 691 authors (median of 2 authors per included publication, interquartile range 1, 4). Over half (54.0%, n = 373) of the included authors had names that were female (Table 2).

**Table 1:** Characteristics of Included Publications on Decolonizing Global Health or Global Health Partnerships (n = 197 publications).


	n (%)

**Article Type**	

Viewpoint/Commentary/Editorial	177 (89.8)

Original Research	20 (10.2)

**Focus of Publication Content**	

Decolonizing Global Health	99 (50.3)

Global Health Partnerships	64 (32.5)

Both	34 (17.2)

**Number of Authors (median, IQR)**	2 (1, 4)

**Author Affiliation Income Bracket**	

High-Income Countries (HIC) Only	138 (70.1)

Low- or Middle-Income Countries (LMIC) Only	15 (7.6)

Both HIC and LMIC	44 (22.3)

Scimago Journal Ranking*	

Top Quartile	138 (72.0)

2^nd^ Quartile	40 (20.8)

3^rd^ Quartile	7 (3.6)

Lowest Quartile	7 (3.6)

**Funding Source**	

High-Income Country Source	45 (22.9)

Low- or Middle-Income Source	5 (2.5)

None	147 (74.6)


* 5 journals did not have listed Scimago Journal Rankings.

**Table 2:** Characteristics of Authors in Publications on Decolonizing Global Health or Global Health Partnerships (N = 691 authors).


	n (%)	*P* VALUE*

**Income Bracket of Listed Affiliation**		<0.001

High-Income Country	519 (75.1)	

Upper-Middle Income Country	50 (7.2)	

Lower-Middle Income Country	93 (13.5)	

Low-Income Country	29 (4.2)	

**Geographic Region of Listed Affiliations**		<0.001

East Asia and Pacific	75 (10.9)	

Europe and Central Asia	170 (24.6)	

Latin America	23 (3.3)	

Middle East	7 (1.0)	

North America	289 (41.8)	

South Asia	28 (4.1)	

Sub-Saharan Africa	99 (14.3)	

Gender of Name**		0.001

Female	373 (54.0)	

Male	293 (42.4)	


* Compares the distribution of author affiliations within each group using the Chi-square test. ** 25 authors had first names that were not assigned a gender using genderize.io.

### Country Income Bracket Distribution of Authors

Over three quarters (75.1%, n = 519) of the authors were affiliated with HICs and only 4.2% (n = 29) were affiliated with low-income countries (Table 2). Publications with author bylines comprised exclusively of authors affiliated with HICs were most common (70.0%, n = 138) followed by publications that included authors affiliated with both HICs and LMICs (22.3%, n = 44). Of the 138 publications that had no authors affiliated with LMICs, 11.6% (n = 16) had acknowledgements that included individuals affiliated with LMICs. Among the 20 original research articles included, 50% (n = 10) had author bylines comprised exclusively of authors affiliated with HICs and 50% (n = 10) had author bylines that included authors affiliated with both HICs and LMICs. The included original research articles were primarily survey studies (75%, n = 15/20).

Only 7.6% (n = 15) of publications on decolonizing global health and global health partnerships had author bylines comprised exclusively of authors affiliated with LMICs. Among the 15 publications with author bylines comprised exclusively of authors affiliated with LMICs, 8 (53.3%) had authors exclusively affiliated with lower-middle income countries, 4 (26.7%) had authors exclusively affiliated with upper-middle-income countries, and 3 (20.0%) had authors affiliated with a combination of upper-middle-income countries, lower-middle-income countries, or low-income countries.

Female and male authors affiliated with HICs were most common among all authors by gender and country affiliation income bracket (78.6%, n = 293/373 and 71.7%, n = 210/293, respectively) (Table 3). Among the 197 included publications, 51.8% (n = 102) had first authors whose names were female and were affiliated with HICs. One third (33.5%, n = 66) of publications had first authors whose names were male and were affiliated with HICs. There were 7.1% (n = 14) that had first authors whose names were female and were affiliated with LMICs and only 4.1% (n = 8) had male first authors affiliated with LMICs. Among the 137 publications with >1 author, 39.4% (n = 54) had last authors who were female and affiliated with HICs, 41.6% (n = 57) had last authors who were male and affiliated with HICs, and only 8.0% (n = 11) had female last authors affiliated with LMICs. Eight percent (8.8%, n = 12) had last authors who were male and affiliated with LMICs.

**Table 3:** Overall Author Distribution by Gender and Income Bracket and Gender and Geographic Region (N = 666 authors)*.


	n (%)	*P* VALUE**

**Income Bracket and Male (N = 293)**		<0.001

High-Income Country	210 (71.7)	

Upper-Middle Income Country	20 (6.8)	

Lower-Middle Income Country	49 (16.7)	

Low-Income Country	14 (4.8)	

**Income Bracket and Female (N = 373)**		<0.001

High-Income Country	293 (78.6)	

Upper-Middle Income Country	28 (7.5)	

Lower-Middle Income Country	37 (9.9)	

Low-Income Country	15 (4.0)	

**Geographic Region and Male (N = 293)**		<0.001

East Asia and Pacific	31 (10.6)	

Europe and Central Asia	76 (25.9)	

Latin America	11 (3.8)	

Middle East	4 (1.4)	

North America	110 (37.5)	

South Asia	14 (4.8)	

Sub-Saharan Africa	47 (16.0)	

**Geographic Region and Female (N = 373)**		<0.001

East Asia and Pacific	43 (11.5)	

Europe and Central Asia	86 (23.1)	

Latin America	11 (2.9)	

Middle East	2 (0.5)	

North America	171 (45.9)	

South Asia	13 (3.5)	

Sub-Saharan Africa	47 (12.6)	


* 25 authors had names that were not assigned male or female by genderize.io. ** Compares the distribution of author affiliations within each group using the Chi-square test.

There was no significant difference in the proportion of publications that had author bylines comprised exclusively of authors affiliated with HICs, LMICs, or both HICs and LMICs and the Scimago Journal Ranking of the journal in which they were published (*P* = 0.178) (Supplemental Table 1).

### Geographic Distribution of Authors

Of the total, 41% (n = 289) of the authors were affiliated with countries in North America and 24.6% (n = 170) were affiliated with countries in Europe and Central Asia. Authors affiliated with countries in the Middle East were least represented (1.0%, n = 7) (Table 2). Nearly one in four authors were females affiliated with countries in North America (24.7%, n = 171/691) (Table 3). Overall, 23.4% (n = 162/691) of all authors were affiliated with Europe and Central Asia and 13.6% (94/691) were affiliated with countries in sub-Saharan Africa (Table 3). Authors affiliated with countries in the Middle East (1.0%, n = 7) and South Asia were least common (4.1%, n = 28).

For first authors of the 197 included publications, 31.5% (n = 62) had female names and were affiliated with countries in North America and 15.7% (n = 31) had male names and were affiliated with countries in North America (Supplemental Table 2). In contrast, only 1.0% (n = 2) publications had first authors who were female and affiliated with countries in South Asia and only 1.5% (n = 3) had male first authors affiliated with countries in South Asia. There were no first authors affiliated with countries in the Middle East. Among last authors for the 137 publications with >1 author, 25.5% (n = 35) were male and affiliated with countries in North America and 21.9% (n = 30) were female and affiliated with countries in North America (Supplemental Table 3). There were no authors affiliated with countries in the Middle East in the last author position.

**Figure 3:**
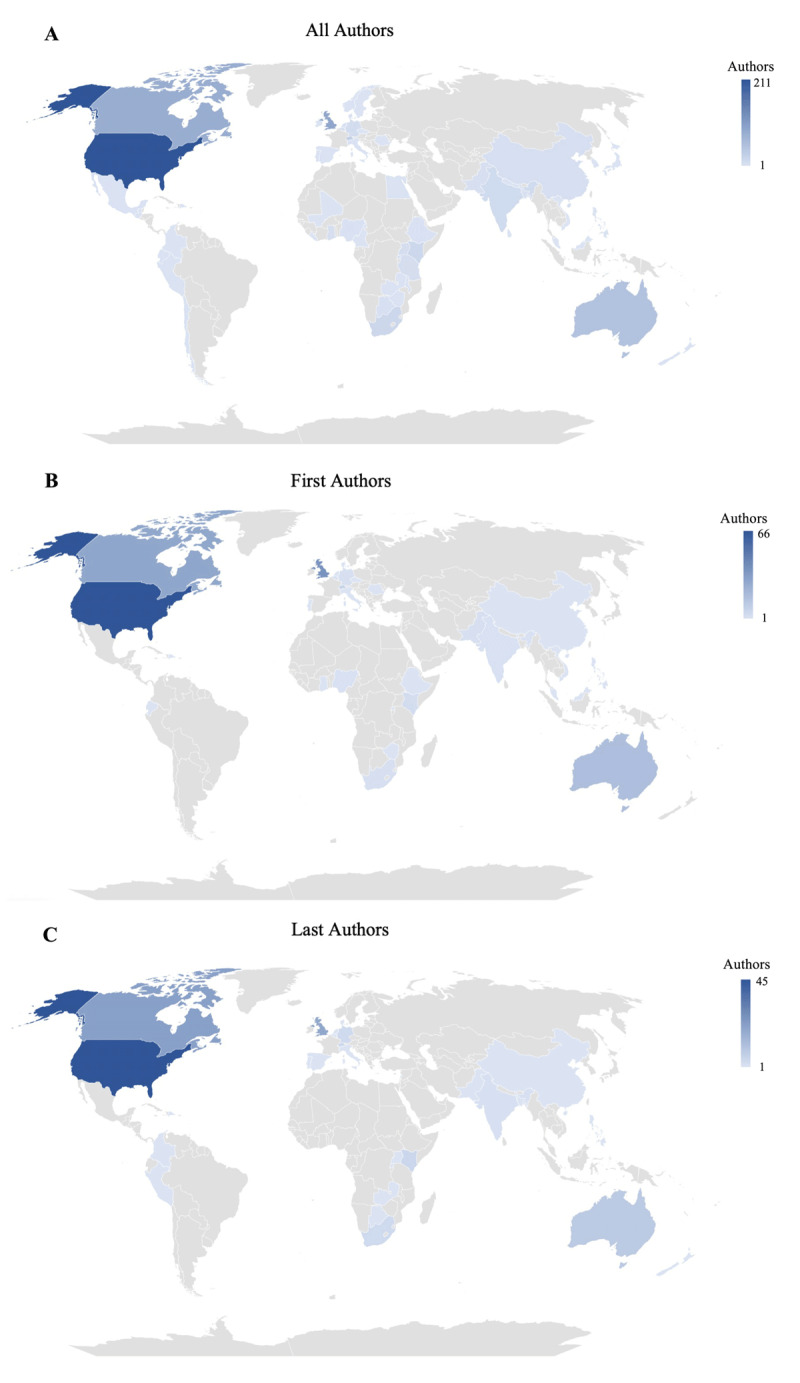
Geographic Distribution of **a)** All Authors, **b)** First Authors, and **c)** Last Authors in Publications on Decolonizing Global Health and Global Health Partnerships.

The geographic distribution of the frequency of authorship in first, last, and all authorship positions is shown in Figure 3. Over half of first authors were affiliated with institutions in just three countries: the United States (33.5%, n = 66), the United Kingdom (19.3%, n = 38), and Canada (14.7%, n = 29). Similarly, among the 137 publications with >1 author, last authors were most commonly affiliated with the United States (32.8%, n = 45), Canada (16.1%, n = 22), and the United Kingdom (14.6%, n = 20).

## Discussion

In our study of 197 publications, there was a large increase in the overall number of publications on decolonizing global health and global health partnerships between HICs and LMICs from 2020 to 2022. The narrative on these publications seems to be primarily driven by opinion-based publications written by authors affiliated with HICs, mostly from European, Central Asian, and North American countries. Female authors affiliated with HICs were the most common authors in these publications.

The movement to decolonize global health has been accompanied by myriad publications that increased from 2020 to 2022. Following several prominent publications on the topic in 2018, this rise in publications on the topic has been noted, but not quantified previously [27,28]. As global health clinicians and researchers who have witnessed the imbalances in benefits derived from collaborations between LMICs and HICs [29,30], we believe that this movement has been long overdue. Additionally, it is imperative that the impact of the decolonizing global health movement be measured. Measuring the impact of this movement may include rigorous studies to assess changes in authorship in publications that report work conducted in LMICs, trends in the geographic location of recipients of grants for global health research [31], and perceptions of equity in global health from investigators affiliated with LMICs. Rigorous research to assess the impact of the decolonizing global health movement is also needed because, to date, publications on this topic have largely been opinion-based.

Though the expansion of publications regarding decolonizing global health and global health partnerships provides insight into nuanced differences in relationships and colonial history in academia, our study demonstrates that the narrative on these topics has, to date, primarily been told by authors affiliated with HICs. This finding aligns with opinion pieces that suggest the voices of representatives in LMICs have largely been absent in these publications [32,33]. Previous studies suggest that more than half of researchers affiliated with LMICs do not know or “have heard little” of decolonizing global health [34], which suggests that the dissemination of this narrative has not been geographically far reaching. Though it is possible that initial publications on these subjects were conceived of by authors in HICs and publications by authors affiliated with LMICs may follow, our findings provide a clarion call for more publications from authors affiliated with LMICs to provide a more balanced narrative of how global health can, and should, be decolonized.

This lack of bilaterality was manifest in our finding that 70% of publications had author bylines comprised solely of authors affiliated with HICs. This number far outpaces the previously reported proportion of publications with author bylines solely comprised of investigators affiliated with HICs in previous studies assessing original research conducted in LMICs, which has ranged from 5–15% [16,17,18,35]. This difference is likely because most of the included publications in this article were opinion-based (i.e., commentaries, editorials, etc.) and prior studies of authorship have focused on publications of original research, which more frequently requires collaboration to successfully execute research. Additionally, journals may have stricter authorship allowances for opinion-based articles, which may have contributed to the relatively low proportion of authors affiliated with LMICs in our study. The perspectives of individuals in LMICs are crucial to gain meaningful contextual insight and to provide robust, specific, and culturally humble solutions to support the decolonizing global health movement and to build meaningful global health partnerships [36]. Though the promotion of the decolonizing global health movement by authors affiliated with HICs may reach a wide audience in high-tier publications, the needs and voices of valued partners affiliated with LMICs should accompany and lead these key messages.

Authors affiliated with HICs whose names were female were most common in publications on decolonizing global health and global health partnerships. Moreover, we found that authors whose names were female were most commonly in the most prominent authorship positions (i.e., first and last authors). These findings differ from those in prior studies that have demonstrated that females are less likely to author invited commentaries [24], to be on editorial boards, or to be first or last authors in publications [22,23,25]. In another study, even if a female author were in the first author position, they were more frequently published in lower-tier journals than publications with males in the first author position [37]. The reasons for the difference in authorship by gender in our study and those of prior studies are unclear. As our study population was largely focused on global health decolonization and partnerships, our findings may imply greater interest in equity among female authors or may relate to the possibility that more females in HICs engage in global health work than males. However, further study is warranted to understand the reasons for the prominence of female authors in this population of publications.

### Limitations

Though we used a robust search query and searched several databases and did not exclude publications based on the language of publication, it is possible that we may not have captured all publications related to decolonizing global health, particularly those that were published in journals not indexed in the included databases. As a large proportion of included publications were opinion-based, funding sources may not have been accurately captured given potential variation in journal requirements for reporting funding for opinion-based publications. Author country affiliations may represent their current country and may not represent their country of origin in the case of individuals who expatriate outside their home country. Additionally, author country affiliation may not fully capture lived experiences that lead to their perspectives of equity or justice in global health. However, results from a previous study suggest there is substantial correlation between an author’s affiliated country and their country of origin [10]. Additionally, despite these limitations, gender and country affiliation are useful proxies for equity within partnerships [38]. Lastly, there are limitations to using software to assign probable genders to authors based on their first name, as gender is a self-reported construct [39]. However, it was not feasible to contact each author to determine their self-reported gender and we expected that non-binary genders would be uncommon.

## Conclusions

The recent rapid expansion of publications on decolonizing global health and global health partnerships has largely been driven by authors affiliated with HICs. There was a marked paucity of publications with authors affiliated with LMICs whose voices provide context and crucial insight into the needs of the decolonizing global health movement. Efforts are urgently needed in the decolonizing global health movement to elevate the voices and lived experiences of authors affiliated with LMICs to provide a more equitable view of the way forward in just global health partnerships.

## Data Accessibility Statement

Data may be made available upon reasonable request to the corresponding author.

## Additional File

The additional file for this article can be found as follows:

10.5334/aogh.4146.s1 Decolonizing Supplement. Supplemental Tables 1 to 3 and Appendix.
