# Supplementary material for: Has Authorship in the Decolonizing Global Health Movement Been Colonized?
Source: Ann Glob Health. 2023 Jun 20;89(1):42. doi: 10.5334/aogh.4146 (PMC10289040; doi:10.5334/aogh.4146)
Supplement: Decolonizing Supplement. — Supplemental Tables 1 to 3 and Appendix. [file agh-89-1-4146-s1.pdf]

Supplemental Table 1. A comparison of Scimago Journal Rank and authorship income bracket composition in publications on decolonizing global health or global health partnerships

	**HIC Only, N=136, n (%)***	**LMIC Only, N=14, n (%)****	**HIC and LMIC, N=42, n (%)*****	***P* Value**
**Top quartile**	94 (69.1)	11 (78.6)	33 (78.6)	0.178
**Second quartile**	32 (23.5)	0 (0.0)	6 (14.3)	
**Third quartile**	6 (4.4)	2 (14.3)	1 (2.4)	
**Bottom quartile**	4 (3.0)	1 (7.1)	2 (4.7)	

*2 publications that only included HIC-affiliated authors did not have Scimago Journal Rank scores.
**1 publication that only included LMIC-affiliated authors did not have a Scimago Journal Rank score.
***2 publications that only included both HIC-affiliated and LMIC-affiliated authors did not have Scimago Journal Rank scores.

Supplemental Table 2. Regions of listed affiliations for first authors of publications on decolonizing global health and global health partnerships (N=190 authors)*

**Female First Author Affiliations**	n	%
East Asia and Pacific	12	6.3
Europe and Central Asia	32	16.8
Latin America	2	1.1
Middle East	0	0.0
North America	62	32.6
South Asia	2	1.1
Sub-Saharan Africa	6	3.2
**Male First Author Affiliations**		
East Asia and Pacific	10	5.3
Europe and Central Asia	25	13.1
Latin America	1	0.5
Middle East	0	0.0
North America	31	16.3
South Asia	3	1.6
Sub-Saharan Africa	4	2.1

*7 of 197 first authors had names that were not assigned a gender.

Supplemental Table 3. Regions of listed affiliations for last authors of publications on decolonizing global health and global health partnerships (N=134 authors)*

**Female Last Author Affiliations**	n	%
East Asia and Pacific	8	6.0
Europe and Central Asia	16	12.0
Latin America	2	1.5
Middle East	0	0.0
North America	30	22.4
South Asia	1	0.7
Sub-Saharan Africa	8	6.0
**Male Last Author Affiliations**		
East Asia and Pacific	6	4.5
Europe and Central Asia	18	13.4
Latin America	1	0.7
Middle East	0	0.0
North America	35	26.1
South Asia	3	2.2
Sub-Saharan Africa	6	4.5

*3 of 137 last authors had names that were not assigned a gender.

Appendix. Search terms used to identify publications related to decolonizing global health and global health partnerships in MEDLINE, Embase, CINAHL, CAB Global Health, and Web of Science

MEDLINE (PUBMED)

(Decoloniz*[tw] or decolonis*[tw] or neocolonial*[tw] or supremacy[tw] or antiracis*[tw] or anti-racis*[tw] or “social justice”[tw] or “partnership”[tw] or “partners”[tw] or "Social Justice"[Mesh:NoExp] or dominance[tw] or domination[tw] or hegemony[tw] or hegemonic[tw] or whiteness[tw])
AND
(“global health”[tw] or "Global Health"[Mesh] or “international health”[tw])

EMBASE
(decoloniz* OR decolonis* OR neocolonial* OR supremacy OR antiracis* OR 'anti racis*' OR ‘partnership’ or ‘partners*’ OR 'social justice' OR dominance OR domination OR hegemony OR hegemonic OR whiteness)
AND
('global health' OR 'international health')

CINAHL
(Decoloniz* or decolonis* or neocolonial* or supremacy or antiracis* or anti-racis* or partnerships or partners* or “social justice” or dominance or domination or hegemony or hegemonic or whiteness)
And
“global health” or “international health”

CAB Global Health
(Decoloniz* or decolonis* or neocolonial* or supremacy or antiracis* or anti-racis* or partnership or partners* or “social justice” or dominance or domination or hegemony or hegemonic or whiteness)
And
“global health” or “international health”

Web of Science
(Decoloniz* or decolonis* or neocolonial* or supremacy or antiracis* or anti-racis* or partnership* or partners* or “social justice” or dominance or domination or hegemony or hegemonic or whiteness)
AND
“global health” or “international health”
